# Importance of Multiple Methylation Sites in *Escherichia coli* Chemotaxis

**DOI:** 10.1371/journal.pone.0145582

**Published:** 2015-12-18

**Authors:** Anna Krembel, Remy Colin, Victor Sourjik

**Affiliations:** 1 Zentrum für Molekulare Biologie der Universität Heidelberg, DKFZ-ZMBH Alliance, Im Neuenheimer Feld 282, D-69120 Heidelberg, Germany; 2 Max Planck Institute for Terrestrial Microbiology & LOEWE Center for Synthetic Microbiology (SYNMIKRO), Karl-von-Frisch-Straße 16, D-35043 Marburg, Germany; University of Illinois at Urbana-Champaign, UNITED STATES

## Abstract

Bacteria navigate within inhomogeneous environments by temporally comparing concentrations of chemoeffectors over the course of a few seconds and biasing their rate of reorientations accordingly, thereby drifting towards more favorable conditions. This navigation requires a short-term memory achieved through the sequential methylations and demethylations of several specific glutamate residues on the chemotaxis receptors, which progressively adjusts the receptors’ activity to track the levels of stimulation encountered by the cell with a delay. Such adaptation also tunes the receptors’ sensitivity according to the background ligand concentration, enabling the cells to respond to fractional rather than absolute concentration changes, i.e. to perform logarithmic sensing. Despite the adaptation system being principally well understood, the need for a specific number of methylation sites remains relatively unclear. Here we systematically substituted the four glutamate residues of the Tar receptor of *Escherichia coli* by non-methylated alanine, creating a set of 16 modified receptors with a varying number of available methylation sites and explored the effect of these substitutions on the performance of the chemotaxis system. Alanine substitutions were found to desensitize the receptors, similarly but to a lesser extent than glutamate methylation, and to affect the methylation and demethylation rates of the remaining sites in a site-specific manner. Each substitution reduces the dynamic range of chemotaxis, by one order of magnitude on average. The substitution of up to two sites could be partly compensated by the adaptation system, but the full set of methylation sites was necessary to achieve efficient logarithmic sensing.

## Introduction

Chemotactic behavior of *Escherichia coli* and other bacteria has been extensively characterized [1–4]. Bacteria generally use temporal comparisons of chemoeffector concentrations to bias their swimming towards favorable directions. The swimming pattern of *E*. *coli* consists of smooth runs that last for ~1 sec and are interrupted by short (~0.1 sec) tumbles. When an increased concentration of a chemoattractant or a decreased concentration of chemorepellent is detected during a run, tumbles are suppressed to ensure a longer run in this direction. Such strategy requires short-term memory that allows bacteria to compare a current concentration of the chemoeffector with that a few seconds ago [5–7].

Chemoattractants are typically sensed through their binding to the periplasmic sensory domains of chemoreceptors that subsequently transmit the signal via a conformational change to the cytoplasmic signaling domain [8]. In *Escherichia coli*, the attractant-induced conformation inhibits activity of the receptor-associated histidine kinase CheA, thus reducing phosphorylation of the downstream response regulator CheY. Because the phosphorylated CheY (CheY-P) acts as a tumbling signal, lower levels of CheY-P yield longer straight runs.

The short-term memory in the chemotaxis system is mediated by the methylation of chemoreceptors by the methyltransferase CheR and their demethylation by the methylesterase CheB [9, 10]. These enzymes add or remove methyl groups at several specific glutamate residues in the cytoplasmic part of the chemoreceptors. Two major chemoreceptors of *E*. *coli*, Tar and Tsr, possess four or five such sites, respectively [11–13]; two of these sites are encoded by glutamines and are subsequently deamidated by CheB [14]. A minor receptor Trg also contains five sites [15], indicating functional importance of multiple methylation sites for chemotaxis. In *E*. *coli*, increased methylation generally promotes active (CheA-activating) receptor conformation, thus offsetting the effect of the attractant stimulation. The methylation kinetics is relatively slow, on time scales of seconds for weak stimuli to minutes for strong stimuli. For cells swimming in a gradient, the level of receptor methylation tracks the level of the attractant stimulation with a delay of a few seconds, creating the memory for temporal comparisons. The receptor methylation system also allows cells to adapt to a constant level of background stimuli, regaining sensitivity to further stimulation. It has been shown that for some attractants *E*. *coli* chemotaxis system maintains roughly constant response sensitivity to a fractional change in concentration (logarithmic sensing) over a wide range of the background levels [16–18]. Such logarithmic sensing is common to many sensory systems, as reflected in a Weber-Fechner law [19] or in its generalization to the time-course of the response called fold-change detection [20, 21].

Consistent with the overall importance of the receptor methylation in chemotaxis, *E*. *coli* receptor mutants with one to four methylation sites substituted by either alanine or aspartate are known to be less efficient in chemotactic spreading on soft-agar plates [22–24]. Notably, these effects are site-specific, meaning that the same substitution at different methylation sites impairs chemotaxis to a different degree. Mutations of one or more of the sites were also shown to affect the dynamics of methylation and demethylation of the remaining sites [25, 26]. Nevertheless, the need of having a specific number of methylation sites and the effects of substitutions at individual sites on the pathway response remained largely uncharacterized. On the other hand, computational models of chemotaxis generally predict that an increased number of methylation sites can enhance precision and robustness of adaptation and to extend the dynamic range of logarithmic sensing [18, 27], but these predictions have not been directly experimentally verified. Here we addressed these questions by studying chemotaxis mediated by mutants of the major *E*. *coli* chemoreceptor Tar in which the methylation sites have been systematically replaced by alanine residues. Combining physiological assays of the chemotactic behavior with studies of the intracellular pathway response, we show that mutations at individual methylation sites have markedly different effects on receptor signaling and chemotaxis. Nevertheless, a full complement of the methylation sites is required for proper logarithmic sensing in chemotaxis, ensuring a sensitive pathway response over a wide range of ligand concentrations.

## Results

### Effects of alanine substitutions on steady-state activity and sensitivity

To investigate the effects of the reduced number of methylation sites on various aspects of chemotaxis, we followed a previously used approach [23] and engineered Tar receptors with all 16 possible combinations of alanine (A) and glutamate (E) residues at the four methylation sites. Although glutamines are more commonly used to mimic methylated glutamates [28, 29], glutamines are deamidated by CheB to produce glutamates and are thus not suitable for in vivo studies in strains that possess the intact adaptation system. In contrast, alanines cannot be further modified, thus permanently reducing the number of glutamates available for methylation.

**Fig 1:**
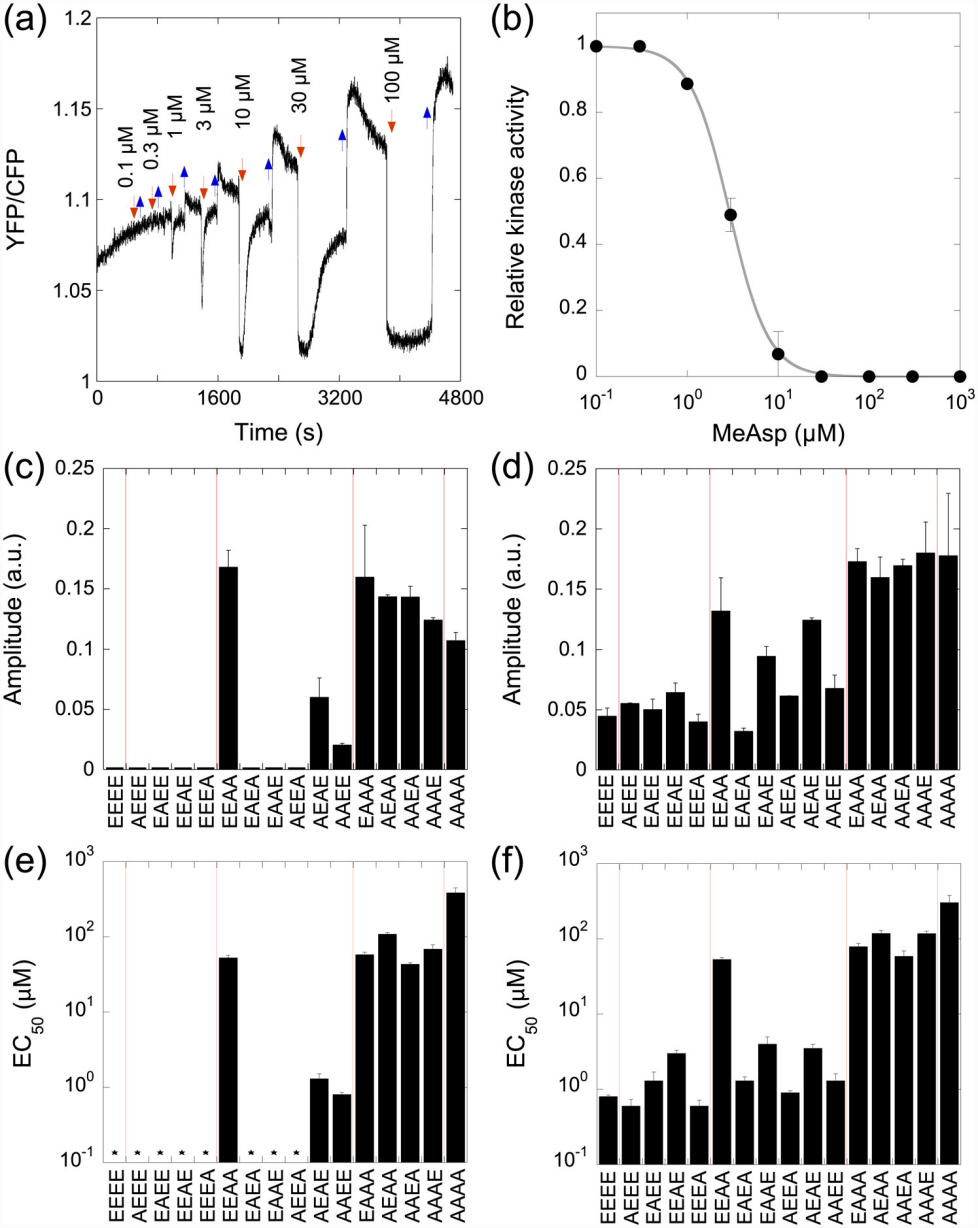
Effects of alanine substitutions on the amplitude and sensitivity of Tar-mediated response. Responses were measured in CheRB+ (VS181) or CheRB^-^ (VH1) receptorless strains expressing Tar from inducible plasmid as a sole receptor. (a) Example of YFP/CFP ratio (proxy for the receptor activity) for a set of step stimulations with increasing concentrations of MeAsp in CheRB^+^ cells expressing Tar^EEAE^. Addition (removal) of attractant is indicated with red (blue) arrows. The amplitude of the response increases until saturation. (b) Dose dependence of the relative kinase activity computed from (a) and fitted using a Hill equation (line) to obtain the value of EC_50_, (MeAsp concentration at the half-maximal response). The change in YFP/CFP ratio (Δ*r*) was normalized to the maximum change in order to evaluate the relative kinase activity as 1 − Δ*r/*Δ*r*_max_ (see text for details). (c,d) Maximal amplitude of the response to an addition of attractant for all modified receptors in CheRB^-^ (c) or CheRB+ (d). (e,f) EC_50_ computed as in (b) for all modified receptors in CheRB^-^ (e) or CheRB+ (f). Asterisks in (e) indicate Tar constructs for which the EC_50_ could not be defined because of zero response (c). Vertical red lines in (c-f) separate groups of receptors with different number of methylation sites.

We have first tested the effects of the alanine substitutions on the ability of Tar to activate the kinase and to mediate responses to its ligand α-methyl-DL-aspartate (MeAsp). Using a previously described FRET assay of the pathway activity, which relies on phosphorylation-dependent interactions between CheY-YFP and CheZ-CFP [30–32], we carried out dose-response measurements of the pathway response in cells expressing engineered Tar as a sole receptor. The cells were stimulated with a step-like addition and subsequent removal of increasing concentrations of MeAsp. The FRET response was quantified as the amplitude of the change in the ratio of the YFP/CFP fluorescence (Fig 1a), which is proportional to the level of CheY phosphorylation and thus to the kinase activity. The resulting dose-response curves (Fig 1b) were used to determine the amplitudes of the response to saturating stimuli as well as the EC_50_ values of the response mediated by individual receptors (Fig 1c–1f). Because saturating stimuli fully inhibit kinase activity even in strains expressing highly modified Tar receptors [29–31, 33], the differences in the response amplitudes reflect the capability of individual receptors to activate the kinase in buffer-adapted cells.

These measurements were performed in two background strains: one possessing the intact receptor modification system (VS181; CheR^+^ CheB^+^) and another lacking this system (VH1; Δ*cheRcheB*). In the VH1 strain, where receptor modification level remains fixed, we observed no measurable response to MeAsp for receptors with zero or one A substitutions as well as for three out of six receptors with two A substitutions (Fig 1c and 1e), suggesting that Tar fixed in these modification states is not able to sufficiently activate the kinase. Similarly, no response was observed in the receptorless VH1 strain expressing no Tar (data not shown). However, higher modification states of Tar showed clear responses, confirming that E to A replacement has an activating effect on Tar. As observed previously for the E to Q substitutions [28, 29, 34], there was a general increase in the EC_50_ values with receptor activity. Such correlation is expected within the theoretical framework used to describe receptor signaling, which postulates that more active receptors are less sensitive to attractants [35]. Notably, the effects of substitutions on the receptor activity were clearly site-specific, with only some of the two-substituted receptors being able to activate the kinase.

In the VS181 (CheRB^+^) strain, responses were observed for all tested receptors, suggesting that an intrinsically low activity of the receptors with low modification increases due to their methylation at the available glutamates. This was indeed confirmed by a direct analysis of Tar methylation using immunoblotting (S1 Fig, panels A and B). The resulting activity and EC_50_ of all receptors with one substitution and most receptors with two substitutions were comparable to those of Tar^EEEE^, suggesting that the methylation system can raise the activity of these receptors nearly independently of the A substitutions. In general, modification on the site 3 had the strongest activating effect on Tar.

### Rate and precision of adaptation

**Fig 2:**
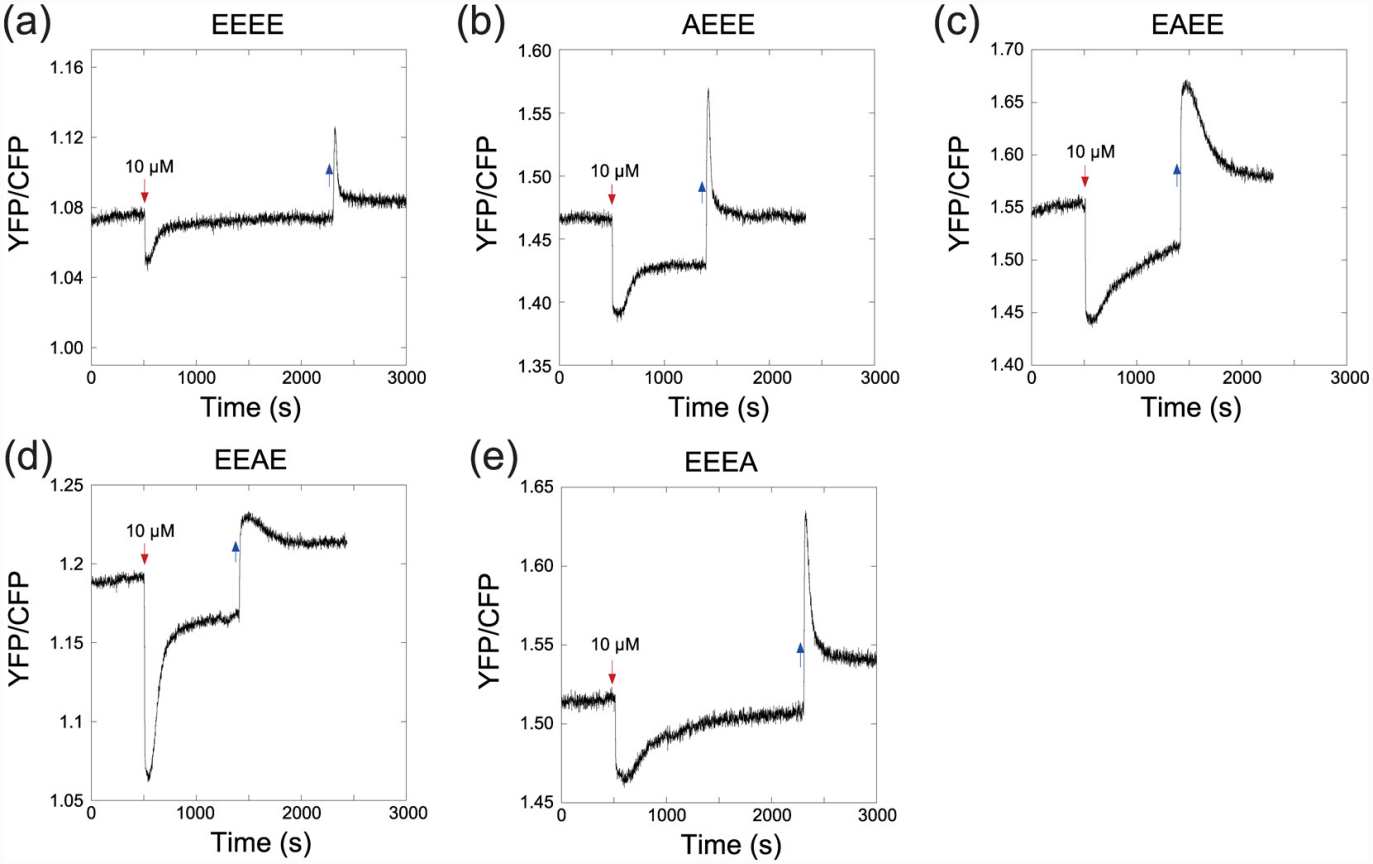
Effects of alanine substitutions on adaptation kinetics. Adaptation kinetics was measured upon stepwise addition (red arrow) and subsequent removal (blue arrow) of 10 μM MeAsp for Tar^EEEE^ (a), Tar^AEEE^ (b), Tar^EAEE^ (c), Tar^EEAE^ (d) and Tar^EEEA^ (e) expressed in VS181 (CheRB^+^) background.

We also observed that alanine substitutions affected the kinetics of adaptation in a site-specific manner (Fig 2). Substitutions at sites 2 or 4 have strongly slowed down adaptation to the addition of attractant (Fig 2c and 2e), whereas mutations at sites 2 or 3 reduced the rate of adaptation to the removal of attractant (Fig 2c and 2d). These results suggest differential effects of alanine substitutions on the rates of CheR and CheB, which mediate adaptation to addition and removal of attractant, respectively. Moreover, all alanine-substituted receptors showed reduced precision of adaptation after stimulation, confirming that the exact balance of the rates of methylation and demethylation is essential for precise adaptation [36, 37]. The effects of multiple alanine substitutions on the rates and the precision of adaptation were even more severe (S2 Fig).

### Dynamic range of the pathway response

**Fig 3:**
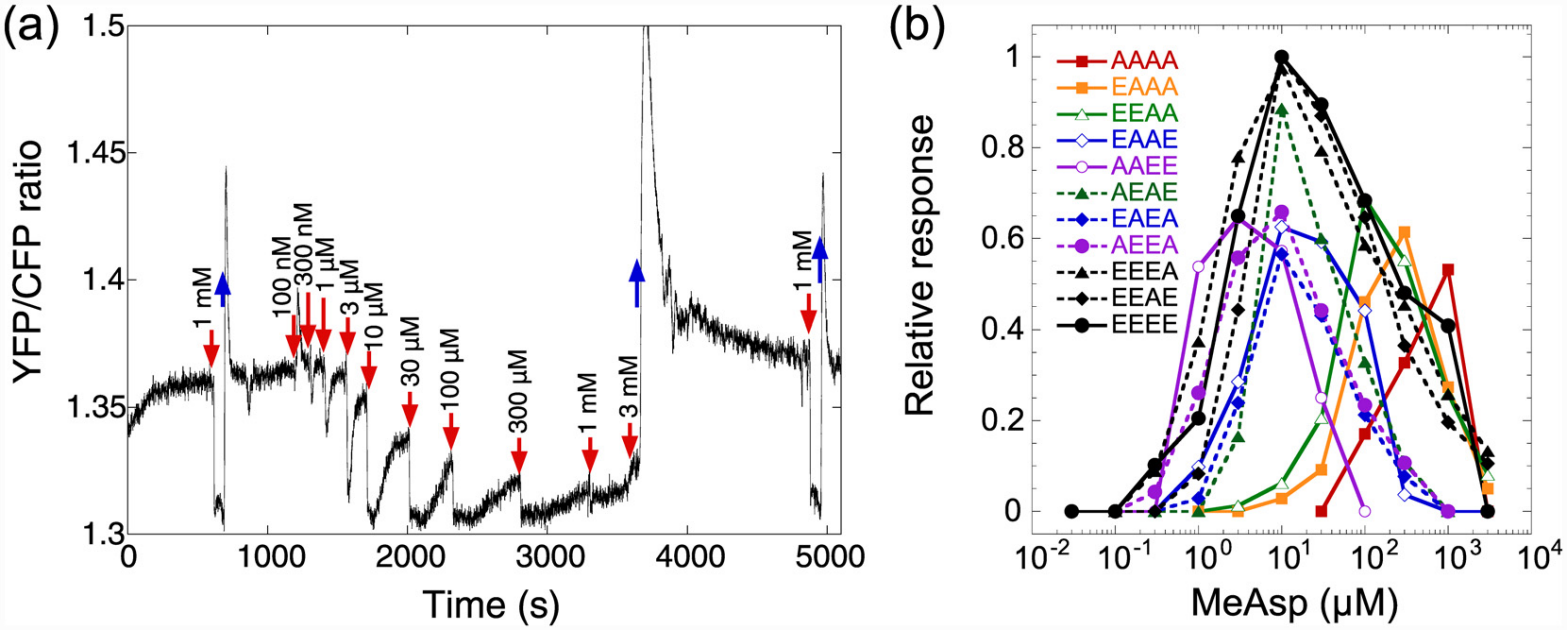
Dynamic range measurement. (a) Example of the FRET measurement for Tar^AAEE^ expressed in VS181 (CheRB^+^) background. Cells were stimulated with sequentially increasing amounts of MeAsp in threefold steps, allowing the activity to adapt between the steps. The response to a saturating stimulus was tested before and after the experiment as a control. (b) Response amplitudes as a function of concentration for VS181 (CheRB^+^) strain carrying indicated receptors.

Another important property of the chemotactic response is its dynamic range, i.e., the range of attractant concentrations over which the system responds to increasing levels of attractant. In the FRET assay, the dynamic range can be measured by stimulating cells with increasing attractant concentrations in approximately fixed-fold steps (three-fold in this case), always allowing cells to adapt before the next subsequent increase [38] (Fig 3a). The lower bound of thus measured dynamic range is determined by the EC_50_ of the response, while the upper bound is either limited by the failure of adaptation to high levels of attractant or by the saturation of the receptors [32, 38].

Consistent with previous analyses performed for wild-type cells [30, 38], the dynamic range of the response mediated by Tar^EEEE^ was broad, spanning nearly four orders of magnitude of MeAsp concentrations (Fig 3b). Substitutions of single alanines had only a modest effect on the width of the dynamic range, narrowing it by approximately half an order of magnitude. The dynamic range response for all zero- and one-substituted receptors peaked at ~10 μM MeAsp. The one-modified receptors also maintained high response sensitivity, with a threefold change in the MeAsp concentration around the peak yielding a saturating response, i.e., completely inhibiting the kinase activity. In contrast, all tested two-modified receptors showed a significantly narrowed dynamic range and lower sensitivity. The dynamic range curves of individual two-substituted receptors were shifted relative to each other, consistent with differences in the EC_50_ that were observed for these receptors (Fig 1f and S1 Table). Substitutions of the third and fourth methylation sites reduced the dynamic range even further. Thus on average, one available methylation site extends the dynamic range of sensing by slightly less than one order of magnitude of ligand concentrations.

### Spreading and chemotaxis in soft agar

**Fig 4:**
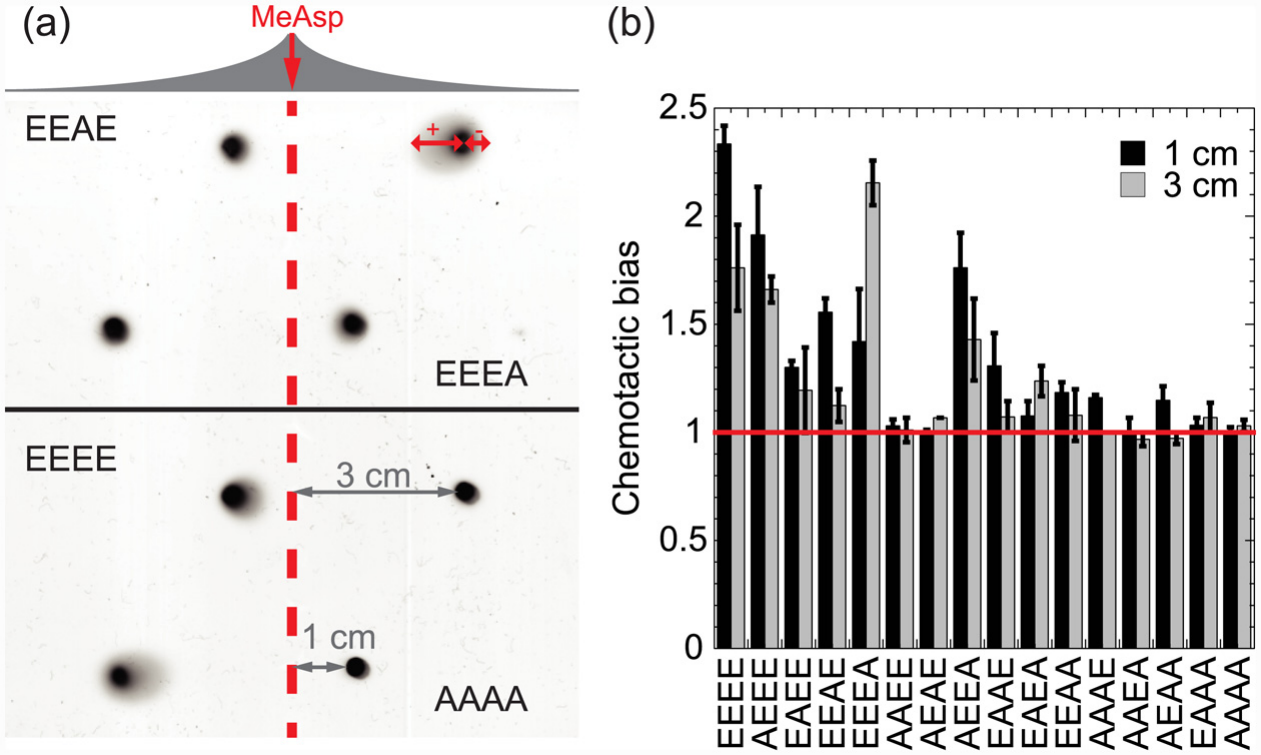
Chemotaxis of alanine-substituted Tar mutants on soft-agar gradient plates. (a) Gradient plate measurement. Gradient of MeAsp was established by applying 0.1 M solution in the middle of the minimal media plate (red dashed line), and allowing the chemical to diffuse. Receptorless UU1250 cells expressing indicated receptors were inoculated at either 1 or 3 cm from the line, and allowed to grow and chemotaxis. Tar^EEEE^ and Tar^AAAA^ were used as positive and negative controls, respectively. Greater colony extension towards the attractant reflects the chemotactic ability of the strain. The chemotactic bias is defined as the ratio of the up-gradient (+) to the down-gradient (-) extension. (b) The chemotactic bias for all receptors and inoculation positions.

The ability of the mutant chemoreceptors to mediate spreading and chemotaxis in a porous medium was tested on minimal media soft-agar plates with a pre-established gradient of MeAsp (Fig 4). In this assay, swimming cells can spread in an undirected fashion through the pores created by the agar, but spreading in the direction of the gradient is enhanced by chemotaxis. Notably, results of this assay are generally consistent with the more frequently used trypton broth (TB) soft agar plates, where cell spreading is enhanced by chemotaxis in self-generated gradients of metabolized amino acids (S3 Fig). However, unidirectional spreading in TB soft agar requires metabolism of the attractant and is sensitive to the average tumbling rate of the cells, and using a pre-established directional gradient of a non-metabolized attractant allows to better disentangle the effects of chemotaxis, motility and metabolism.

The chemotactic bias in the gradient plate assays was determined as the ratio of cell spreading up (+) and down (-) the gradient, with values above unity indicating positive chemotaxis. This allowed us to average out the variable spreading efficiency for the different strains, and thus to specifically determine the sensitivity of each strain to MeAsp. Cells expressing Tar^EEEE^ showed chemotactic bias of approximately two, whereas the negative control Tar^AAAA^ was spreading symmetrically up and down the gradient, resulting in a chemotactic bias of one (i.e., no chemotaxis). Among the one-substituted receptors, Tar^EEEA^ and Tar^AEEE^ showed chemotactic bias similar to that of Tar^EEEE^, whereas Tar^EEAE^ and Tar^EAEE^ showed a significantly reduced response, consistent with these substitutions having largest effects on the EC_50_ of the response (Fig 1) and on the adaptation kinetics, particularly for adaptation to negative stimuli (removal of attractant; Fig 2). Interestingly, the response of Tar^EEAE^ was particularly reduced at a higher distance from the gradient source (i.e., in the lower concentration range), whereas the response of Tar^EEEA^ was reduced in the higher concentration range. This is consistent with our observation that the EC_50_ of Tar^EEAE^ is higher than the one of Tar^EEEE^, whereas the one of Tar^EEEA^ is lower. Three-substituted receptors were unable to convey any chemotactic response, consistent with the higher basal activity and EC_50_ of these receptors. The only two-substituted receptors capable of a (partially impaired) chemotactic response were Tar^AEEA^ and to a lesser extent Tar^EAAE^. We further observed that general unidirectional spreading of bacteria was reduced at high concentrations of attractant, in particular for substituted receptors. This might be explained by their inability to precisely adapt in this concentration range, which would lower the tumbling rate and partly impair spreading on soft agar.

### Chemotactic behavior in a liquid

To further investigate the effects of alanine substitutions on the chemotactic behavior of free-swimming cells in a liquid, we used a recently described assay [39, 40]. Here the bacteria are exposed to linear gradients of MeAsp of a constant relative strength over a range of background concentrations and their averaged chemotactic drift (*v*_*ch*_) as well as the average swimming speed (*v*_0_) are analyzed. Additionally, the fraction of swimming bacteria in the field of view (*α*) is determined in each experiment. The chemotactic bias is subsequently defined as *v*_*ch*_/*αv*_0_, which is zero for non-responding cells and unity for a population where all cells swim directly up the gradient.

**Fig 5:**
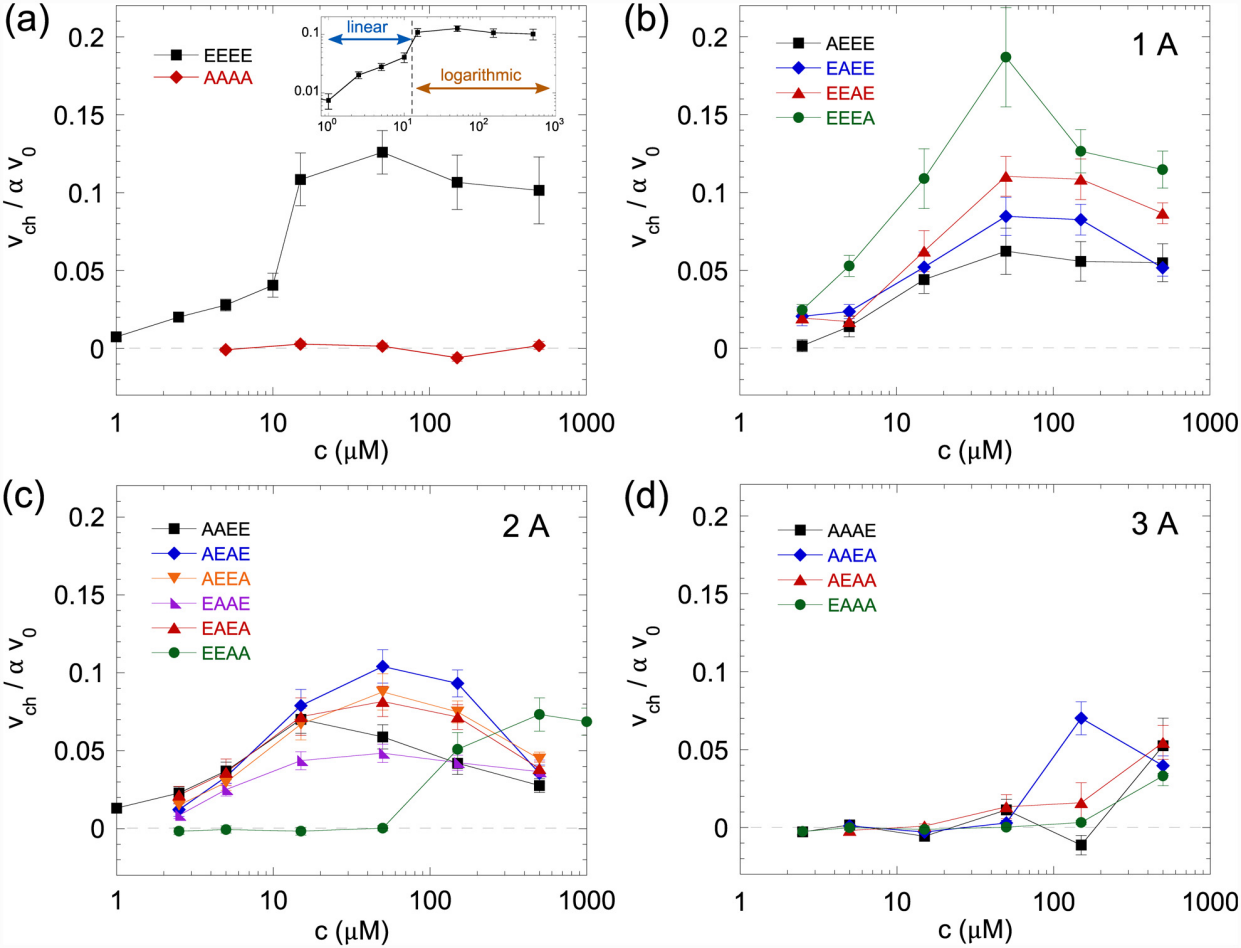
Chemotactic bias of alanine-substituted Tar in gradients. The chemotactic bias (*v*_*ch*_/*αv*_0_) of swimming cells was assayed in gradients of MeAsp with constant relative steepness (∇*c*/*c*) but varying background concentration *c*. The chemotactic bias is shown for unmodified and fully modified (a), one-modified (b), two-modified (c) and three-modified (d) Tar expressed in UU1250 background.

Cells expressing Tar^EEEE^ showed an increase in the chemotactic bias at low concentrations of attractant followed by a plateau (Fig 5a). This is consistent with results that were previously obtained for the wild-type cells with a full complement of receptors and suggests an absolute gradient sensing regime followed by a logarithmic gradient sensing regime. In contrast, Tar^AAAA^ strain was fully unable to respond to gradients in the range of background concentrations investigated (Fig 5a). Cells expressing one-modified receptors in general showed chemotactic bias comparable to that of the Tar^EEEE^ strain (Fig 5b), and over a limited range of concentrations the chemotactic efficiency was even higher for the Tar^EEEA^ strain. However, logarithmic gradient sensing was strongly restricted or entirely abolished for these receptors. The only apparent exception was the Tar^AEEE^ strain, which did show logarithmic sensing in a rather broad range of concentrations, but a maximal chemotactic bias significantly lower than for Tar^EEEE^. The two-modified strains showed essentially no logarithmic sensing (Fig 5c), although the maximal response of the Tar^AEAE^, Tar^AEEA^ and Tar^EAEA^ strains were comparable to that of the Tar^EEEE^ strain in a narrow range of MeAsp concentrations. Consistent with the FRET measurements of the dynamic range, this maximum was reached at different MeAsp concentrations for different substitutions. The Tar^EEAA^ strain was particularly affected and also did not show any response at low attractant concentrations. The Tar^EAAE^ showed a strong defect in the chemotactic bias, but a fairly large range of logarithmic sensing—consistently with having the largest dynamic range of the two-substituted strains. The strains with three alanine substitutions showed no response to the chemoattractant, except for the highest tested concentrations (Fig 5d), whereby the Tar^AAEA^ strain had the highest sensitivity. As expected, strains with highly modified Tar also had lower average swimming speed because of their high tumbling rate (S4 Fig).

## Discussion

The receptor methylation system serves several essential functions in bacterial chemotaxis, but importance of having a particular number of methylation sites remained unclear. Here we investigated the effects of different combinations of alanine substitutions at all four methylation sites of the major *E*. *coli* receptor Tar on its ability to mediate attractant responses. The alanine substitutions had multiple effects on the response profile of Tar measured by FRET assay. These substitutions had a generally activating effect on the kinase activity, although the activation by alanines is apparently less efficient than activation mediated by methylated glutamates or by glutamines [29, 33]. The extent of activation was site-specific, with site 3 having the largest effect. We also observed correlation between activity and EC_50_ of the response for different mutants, similar to the correlation that was previously observed for glutamine-substituted receptors [29, 33]. Notably, for two or fewer substitutions the adaptation system was largely capable of adjusting the receptor activity to roughly the same level as for Tar^EEEE^, confirming the robustness of the adaptation system against perturbations [37]. Nevertheless, the rates of methylation and demethylation of the remaining glutamates were affected by alanine substitutions. This is apparently consistent with a previously reported interplay between methylation at the neighboring sites [25, 26]. Here, sites 2 and 3 had pronounced effects on the demethylation kinetics, whereas sites 2 and 4 strongly affected methylation kinetics.

Another major effect of receptor modifications was to reduce the range of attractant concentrations for which the receptor is capable of mediating a response. This reduction of the dynamic range became more pronounced with an increasing number of substitutions. It could be explained on one hand by a higher response threshold (EC_50_) and on the other hand by an earlier saturation of the receptors methylation sites [38]. On average, one alanine substitution reduces the dynamic range by slightly less than an order of magnitude.

These effects on the activity, sensitivity, adaptation kinetics and dynamic range directly translate into reduced chemotactic response of the population in gradients that were established either in soft agar or in the liquid. Overall, the soft-agar assay where bacteria navigate through the agar pores to follow chemical gradients appears to be more stringent. Here, already alanine substitutions at one of the sites—particularly at sites 2 and 3 –result in severe defects in chemotaxis. Out of two-substituted receptors, only Tar^AEEA^ shows significant chemotaxis in this assay. The apparent importance of sites 2 and 3 in the soft-agar assay agrees with previous observations [22–24] and might be related to the effects of these sites on the adaptation kinetics and on the steady state activity of Tar.

The gradient assay in the liquid provided a more quantitative measure of chemotaxis over a range of attractant concentrations. The population level chemotactic velocity (drift velocity) in the gradient was again affected in all mutants, but in a different way. In this assay, one- and two-substituted receptors—including those that showed poor or no chemotaxis in the soft-agar assay—showed chemotaxis comparable to that of the wild type. However, with a sole exception of Tar^EEEA^, the chemotactic velocity was reduced by alanine substitutions. The maximal reachable response apparently correlates with the precision of the adaptation (S5 Fig). This makes sense because an imprecise adaptation leads to a background activity far off the optimal activity for the motor response [41–43]. Even more importantly, most mutants showed a reduced range of attractant sensing, consistent with FRET measurements of the dynamic range. The overall comparison of the one- and two-substituted receptors with the wild type shows that individual mutants show no clear plateau in the chemotactic velocity at high ligand concentration that is characteristic of logarithmic sensing. The only apparent exception is the response of Tar^AEEE^, which nevertheless has markedly lower amplitude compared to the wild-type response. Our results thus demonstrate that all four methylation sites are essential to ensure sensitive logarithmic sensing in bacterial chemotaxis.

While the absolute majority of chemotactic bacteria encode the CheR-CheB adaptation system, many bacteria also possess additional adaptation systems, which can exhibit complex cross-regulation with the CheR-CheB system [44, 45]. Moreover, not only the number of methylation sites but also the effects of methylation on the pathway activity might differ between receptors and bacterial species [46]. It thus remains to be seen how our results obtained for the simpler adaptation system of *E*. *coli* can translate to more complex chemotaxis pathways.

## Materials and Methods

### Strain construction and growth conditions

For all FRET experiments, *E coli* strain VS181 (lacking all chemoreceptors as well as native *cheY* and *cheZ* genes) or its derivative VH1 that additionally lacks *cheR* and *cheB* genes were used. Plasmids used in this study are described in S2 Table. pVS88 plasmid expressing the CheY-YFP/CheZ-CFP FRET pair from a bicistronic construct has been described before [47]. Alanine substitutions in Tar were introduced by PCR and similar expression of all mutants was verified using immunoblotting (S1 Fig panel C). Cells were grown at 34°C and 275 rpm in tryptone broth (TB; 1% tryptone, 0.5% NaCl) with appropriate antibiotics (100 μg ml^-1^ ampicillin; 17 μg ml^-1^ chloramphenicol). Cultures were diluted 1:20 from overnight cultures and grown to an optical density at 600 nm (OD_600_) of 0.6 in TB medium supplemented with antibiotics and 50 μM isopropyl-β-D-thiogalactopyranoside (IPTG) to induce expression of the CheY-YFP/CheZ-CFP FRET pair from pVS88 and 2 μM sodium salicylate to induce expression of the respective mutant Tar chemoreceptor. Cells were resuspended in tethering buffer (10 mM KPO_4_, 0.1 mM EDTA, 1 μM methionine, 10 mM lactic acid, pH 7) and kept at 4°C for at least 30 minutes to decrease the metabolic activity of the cells.

For all population assays, *E coli* strain UU1250, a derivative of strain RP437 lacking all chemoreceptors but with native *cheY* and *cheZ* proteins, was grown following the same protocol as the FRET strain, with only 2 μM salicylate as inducer, such that the induction level of the Tar receptor is the same in all experiments. For the free-swimming assay, the cells were resuspended in motility buffer (10 mM KPO_4_, 0.1 mM EDTA, 67 mM NaCl, pH 7) supplemented with 1% wt glucose.

### Dose response measurements and determination of EC_50_

FRET measurements were performed as described before [30, 31, 38, 48, 49] on custom-modified Zeiss Axiovert 200 or Axio Imager.Z1 fluorescence microscopes. Cells were harvested by centrifugation (3200 x *g* for 5 min), washed once with 10 ml tethering buffer (10 mM KPO_4_, 0.1 mM EDTA, 1μM methionine, 10 mM lactic acid, pH 7.0), resuspended in 9 ml tethering buffer and stored at 8°C. For FRET experiments, cells were attached to a polylysine-coated coverslip and kept under a constant flow of tethering buffer at a rate of 300 μl/min in a flow chamber. To add or remove attractant, the attached syringe pump was stopped briefly. Attractant concentrations used to stimulate the buffer-adapted cells were increased in threefold steps, and in between always changed back to attractant-free buffer. The sample was excited at 436/20 nm by 120W EXFO X-Cite^®^ 120 lamp (Axio Imager.Z1) or by 75W Xenon lamp (Axiovert 200) that were attenuated 500-fold or 550-fold, respectively, with neutral density filters. Fluorescence of a monolayer of 300–500 cells was continuously recorded in the cyan and yellow channels using photon counters with a 1.0 s integration time.

Resulting responses were plotted as a dose-response curve and fitted using the Hill equation, where the concentration that causes 50% reduction of the kinase activity, defined as the EC_50_, could be identified.

### Dynamic range measurements

FRET measurements were performed as above, but attractant concentrations used to stimulate a response were increased in threefold steps, and the next higher concentration was only applied after adaptation of cells to the previous concentration, monitored by the time course of the YFP/CFP ratio. For adaptation-defective strains, adaptation was allowed to proceed for 15 minutes of incubation, within the physiological range of adaptation times. The relative responses to the different MeAsp concentrations were determined by calculating the ratio of the respective response to a response to a full saturating stimulus, measured before or after the dynamic range measurement.

### Adaptation kinetics measurements

Cells expressing the respective receptor as well as the FRET reporter pair were kept under a constant flow of buffer for 30 minutes to monitor the steady-state activity of the kinase CheA before the attractant was added for the same time. Upon stimulation, an almost instantaneous decrease of the YFP/CFP ratio indicates inactivation of the kinase. Recovery of the kinase activity by adaptation was determined by analyzing the slope of the adaptation curve.

### Immunoblotting

Strains VH1 or UU1250 expressing alanine-modified receptors were grown as above, resuspended in tethering buffer and where indicated prestimulated with 100 μM MeAsp. Cells were lysed in Laemmli buffer by heating for 5 min at 95°C. Proteins were separated using an 8% SDS polyacrylamide gel of either 12 cm (VH1 strain) or 40 cm (UU1250 strain) length. Proteins were transferred onto a nitrocellulose membrane by wet blotting; the membrane was blocked by incubation with 5% skim milk solution in TBST (2.5 mM Tris, 15 mM NaCl, pH 7.5, 0.1% Tween) for 30 minutes. Receptors were detected using incubations with a 1:5000 dilution of the polyclonal rabbit α-Tar antibody in 1% skim milk solution and the secondary goat α-rabbit IRDye800 (1:5000 dilution in 1% skim milk solution) for 45 minutes each. After a final triple wash in TBS (2.5 mM Tris, 15 mM NaCl, pH 7.5), the membranes were imaged by enhanced chemoluminescence using an Odyssey imager (LI-COR).

### TB soft agar plates

To test the spreading of the generated strains, a soft agar based swarming assay was performed. Therefore, a melted mixture of 100 ml TB and 0.25% agar with respective antibiotics and inducers was poured into a square petri dish. After solidifying, 2 μl of prepared cells were spotted onto the surface of the agar. Plates were incubated at 34°C for around 16 hours, a photo was taken afterwards with Nikon D5200 camera and evaluated using ImageJ software.

### Soft agar gradient plates

For MeAsp gradient plates, 0.25% agar was melted in 100 ml of Minimal A Medium (10.5 g/l K_**2**_HPO_**4**_, 4.5 g/l KH_**2**_PO_**4**_, 1 g/l (NH_**4**_)_**2**_SO_**4**_, 0.5 g/l Na-Citrate×2H_**2**_O), supplemented with 1 ml 20% glycerol, 100 μl 1M MgSO_4_, 800 μl amino acid mix (5 mg/ml of each L-threonine, L-methionine, L-histidine, L-leucine**)** and 200 μl thiamine solution (50 mg/ml) and poured into a square petri dish with respective antibiotic and inducer. The MeAsp gradient was applied after solidifying of the agar by pipetting 12×10 μl 0.1 M MeAsp solution in a vertical line onto the agar surface. Plates were stored at 4°C overnight to allow the establishment of a uniform MeAsp gradient before spotting prepared cells (2μl) onto them with varying distances to the stimulus source. Plates were then incubated at 34°C for around 20 hours and a photo was taken.

### Collective free-swimming gradient response

The complete protocol for measuring the chemotaxis bias is described in [40]. In short, glass hand-made chemotaxis chambers, consisting of two reservoirs, linked via a small channel (lengths L = 1.6–2.4 mm, widths w = 0.8–1.5 mm), containing suspended bacteria along with respectively a concentration c = 0 and c = c_0_ of MeAsp were prepared. After 2 hours, a stable linear gradient of chemoattractant is formed in the channel (see S6 Fig), to which the cells respond. The relative gradient $\left(\frac{1}{c}\frac{\partial c}{\partial x}\right)$ is set by the fixed geometry (length) of the channel and constant for all experiments, while the average background concentration in the middle of the chamber *c* = *c*_0_/2 was varied. The response of each strain to the gradient was recorded, for a wide range of background concentrations, in the middle of the channel using video-microscopy (10x objective under phase contrast illumination, Mikrotron 4CXP camera running at 100 frames per seconds for 100 seconds, with a 717 x 717 μm^2^–512 x 512 px^2^ –field of view, focal plane halfway through the 135 ± 5 μm sample’s depth). A high throughput computer analysis of the films yielded the average chemotactic velocity of the population v_ch_, the population averaged swimming speed of the swimming cells v_0_ and the fraction of swimming cells, *α*, which enables to estimate the chemotactic bias *v*_*ch*_/*αv*_0_.

## Supporting Information

S1 Fig Immunoblotting experiments of Tar methylation. (PDF)

S2 Fig Measurement of adaptation kinetics in 3-substituted receptors. (PDF)

S3 Fig TB swarm plates. (PDF)

S4 Fig Average swimming speed in motility buffer. (PDF)

S5 Fig Quantification of the adaptation half time and precision. (PDF)

S6 Fig Scheme of the microstructured device for liquid gradient assay. (PDF)

S1 Table EC_50_ values of different Tar mutants. (PDF)

S2 Table Plasmids used in this study. (PDF)
