# Supplementary material for: Importance of Multiple Methylation Sites in Escherichia coli Chemotaxis
Source: PLoS One. 2015 Dec 18;10(12):e0145582. doi: 10.1371/journal.pone.0145582 (PMC4684286; doi:10.1371/journal.pone.0145582)
Supplement: S1 Fig — (PDF) [file pone.0145582.s001.pdf]

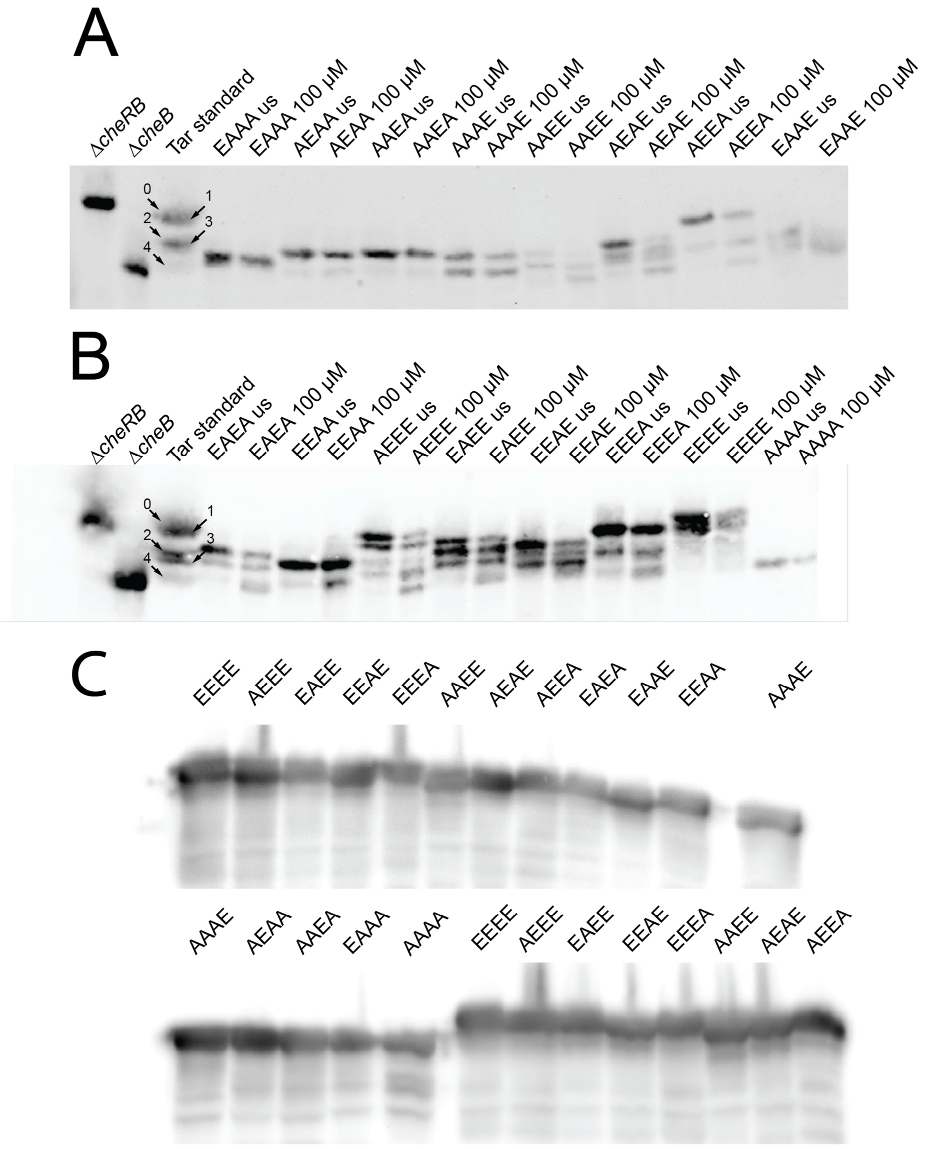

S1 Fig. Immunoblot analysis of alanine-substituted Tar mutants. Tar protein bands were obtained by SDS-PAGE and immunoblotting with a Tar-specific antibody. (A,B) Tar alanine mutants were expressed in UU1250 ($CheR^{+}B^{+}$), cells were harvested and either left unstimulated in the buffer (us) or stimulated with 100 µM MeAsp for 20 minutes, as indicated. Cells were lysed by boiling samples in Laemmli buffer at 95°C. Higher modified receptors (either alanine-substituted or methylated at glutamates) show an increased mobility on the gel, although the effects are partly site-specific. As controls, Δ*cheRB* and Δ*cheB* strains both expressing Tar^{EEEE} were used, which should have Tar in unmodified or fully-modified states, respectively. As Tar standard, samples prepared from Δ*cheRB* strain expressing Tar with fixed numbers of Q residues (as indicated by arrows) was used. (C) Tar alanine mutants were expressed in Δ*cheRB* cells and visualized as above, except that samples were separated on a smaller gel so that differences in Tar mobility are not resolved but protein levels could be compared more directly.
