# Supplementary material for: Importance of Multiple Methylation Sites in Escherichia coli Chemotaxis
Source: PLoS One. 2015 Dec 18;10(12):e0145582. doi: 10.1371/journal.pone.0145582 (PMC4684286; doi:10.1371/journal.pone.0145582)
Supplement: S2 Fig — (PDF) [file pone.0145582.s002.pdf]

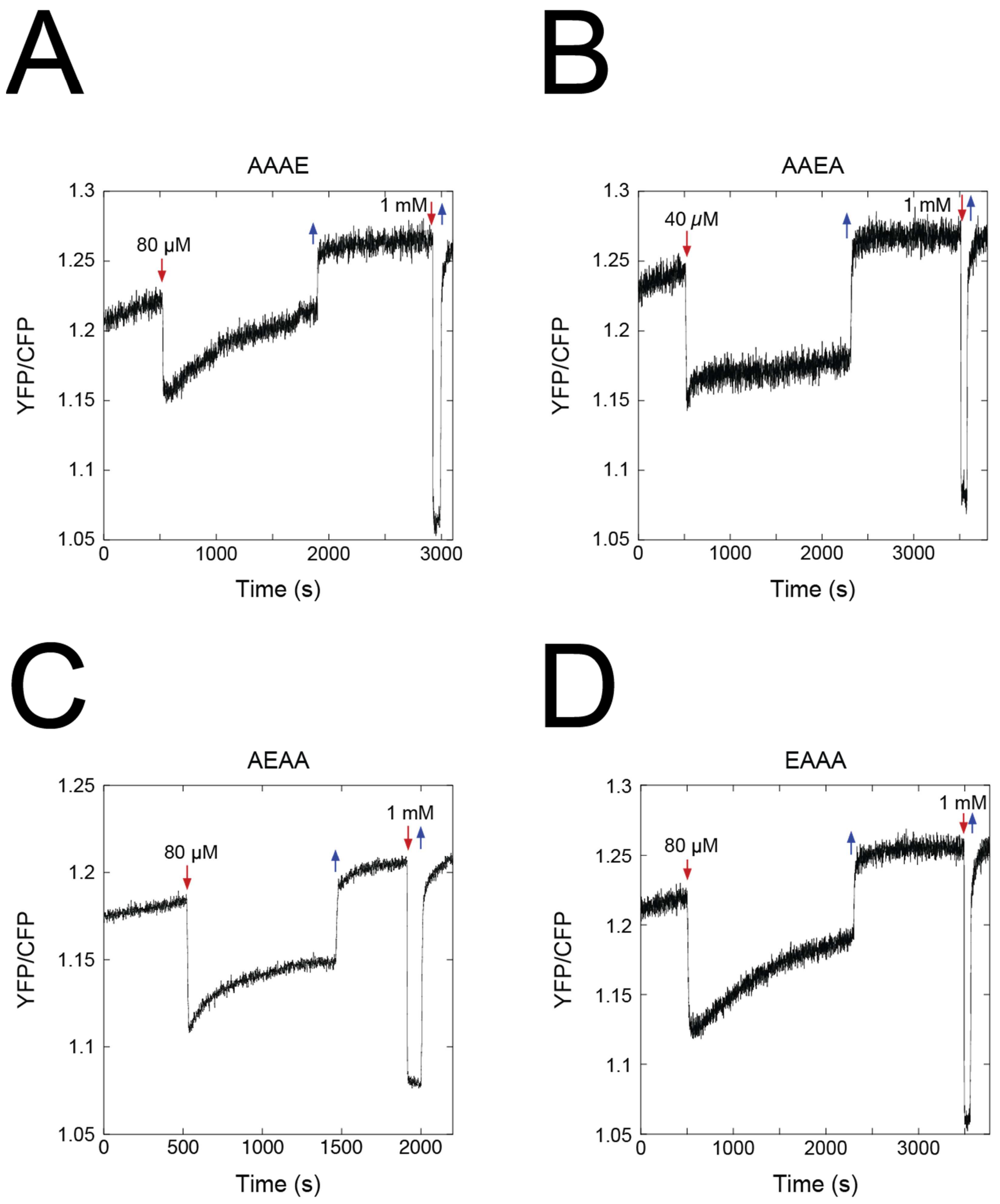

S2 Fig. Measurement of adaptation kinetics in 3-substituted receptors. Adaptation kinetics measurement by FRET for VS181 cells expressing three-substituted receptors AAAE (A), AAEA (B), AEAA (C), EAAA (D). Cells were stimulated by a saturating addition of 40 μM (B) or 80 uM (A,C,D) of MeAsp. Red down and blue up arrows indicate the addition or removal of attractant, accordingly. A control for the maximal (saturating) response is made by exposing cells briefly to 1 mM MeAsp.
