# Supplementary material for: Importance of Multiple Methylation Sites in Escherichia coli Chemotaxis
Source: PLoS One. 2015 Dec 18;10(12):e0145582. doi: 10.1371/journal.pone.0145582 (PMC4684286; doi:10.1371/journal.pone.0145582)
Supplement: S3 Fig — (PDF) [file pone.0145582.s003.pdf]

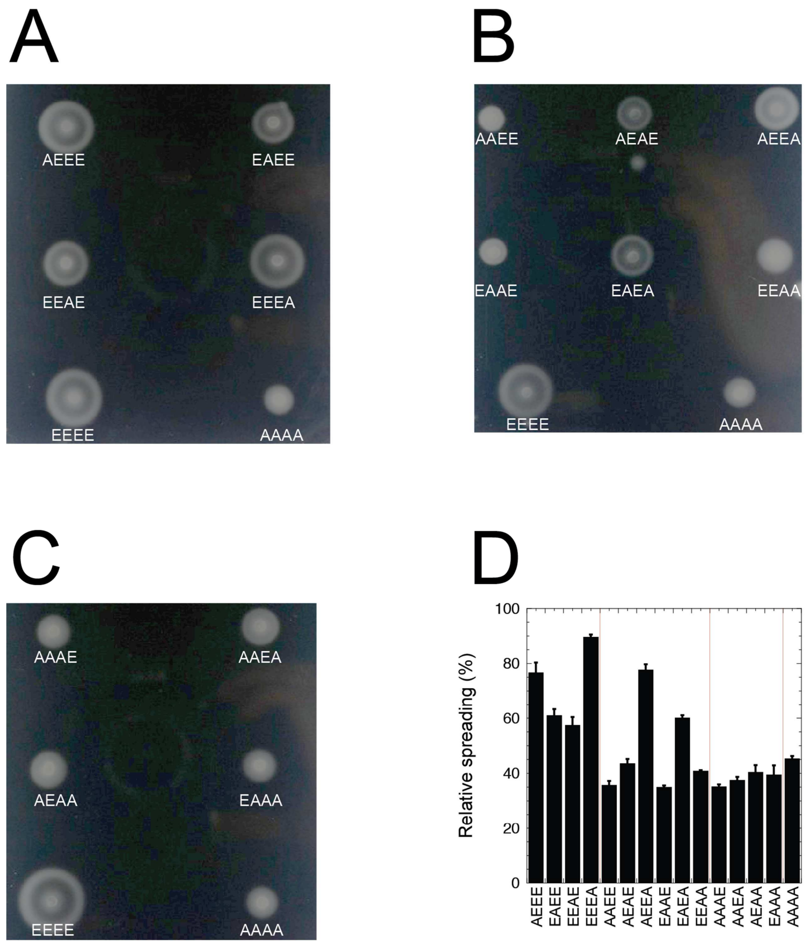

S3 Fig. TB soft agar plates. Examples of TB swimming agar plates for 1-substituted (A), 2-substituted (B) and 3-substituted (C) receptors. The extent of the propagation reflects a combination the ability to move through the soft agar and to respond to a self-generated gradient of nutrients. (D) The averaged radius of propagation (normalized by the value for Tar^{EEEE}).
