# Supplementary material for: Importance of Multiple Methylation Sites in Escherichia coli Chemotaxis
Source: PLoS One. 2015 Dec 18;10(12):e0145582. doi: 10.1371/journal.pone.0145582 (PMC4684286; doi:10.1371/journal.pone.0145582)
Supplement: S4 Fig — (PDF) [file pone.0145582.s004.pdf]

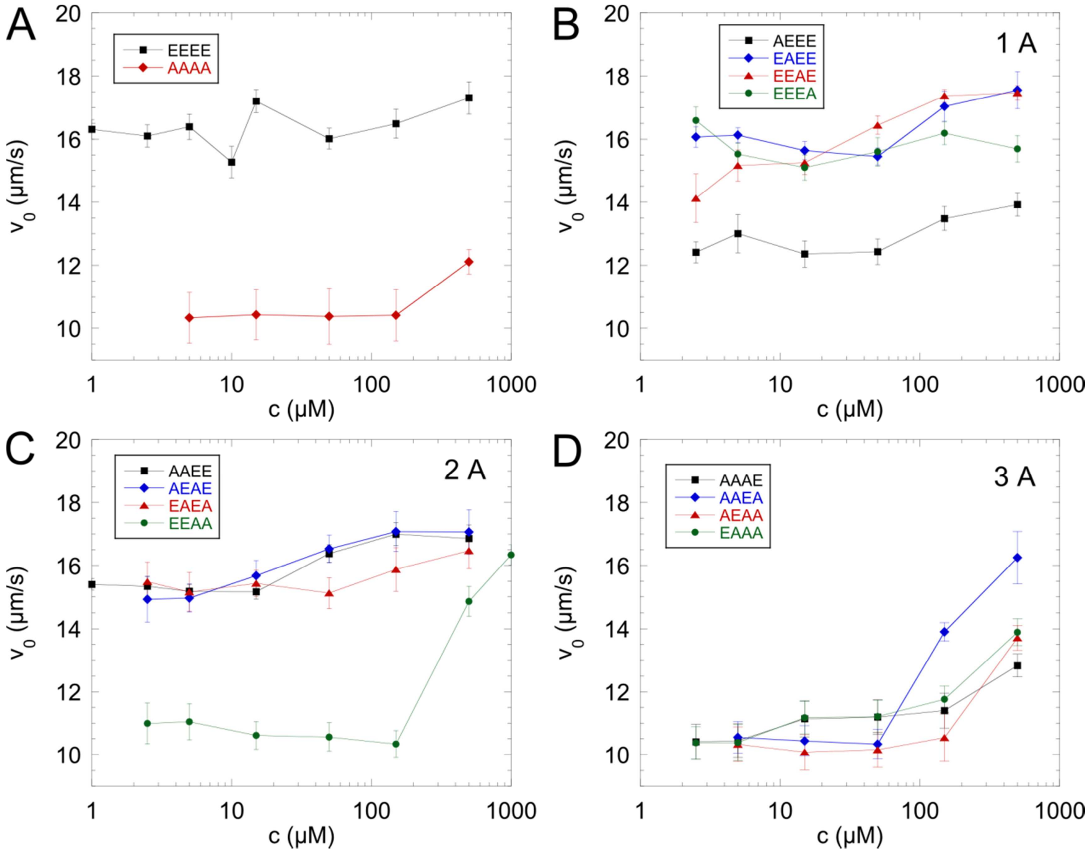

S4 Fig. Average swimming speed in motility buffer. Swimming speeds in liquid motility medium as a function of the background concentration of MeAsp for cells expressing indicated receptors. Here swimming speed was analyzed by Differential Dynamic Microscopy as cells respond to linear gradients of MeAsp (see Fig. 5). (A) Fully intact and fully modified receptors. (B) One-modified receptors, (C) Two-modified receptors, (D) Three-modified receptors. For the AAAA, EEAA and all three modified strains, the low values of the velocities correspond to cells with a high tumbling rate.
