# Supplementary material for: Importance of Multiple Methylation Sites in Escherichia coli Chemotaxis
Source: PLoS One. 2015 Dec 18;10(12):e0145582. doi: 10.1371/journal.pone.0145582 (PMC4684286; doi:10.1371/journal.pone.0145582)
Supplement: S5 Fig — (PDF) [file pone.0145582.s005.pdf]

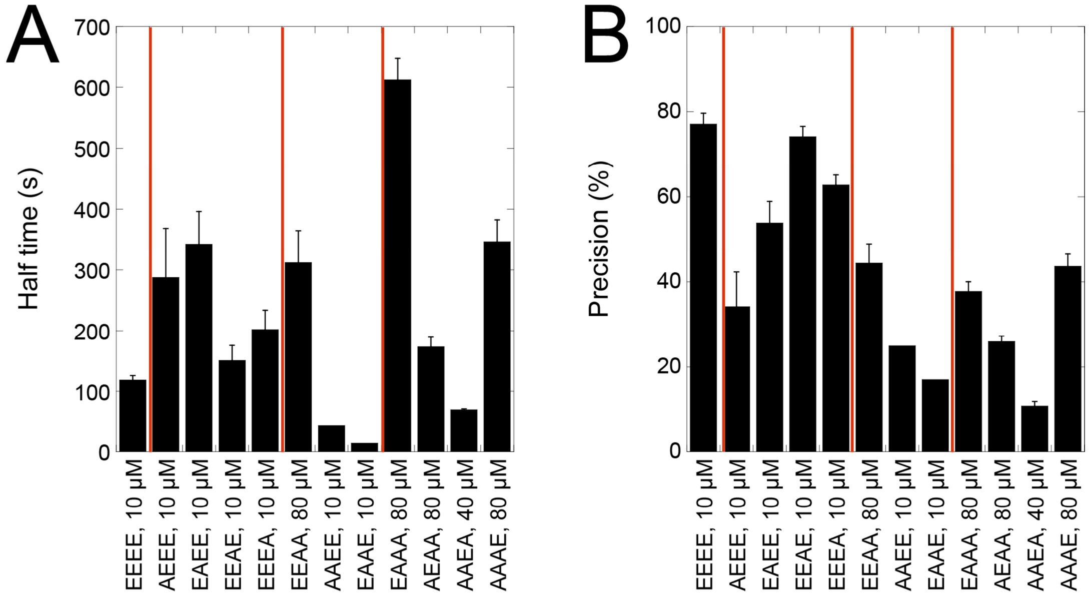

S5 Fig. Quantification of the adaptation half-time and precision. Half times (A) and precision (B) of adaptation to an addition of MeAsp to cells adapted in tethering buffer, measured as in Figure 2 and Figure S2. The amplitude of the response is indicated in the legends. The half time tends to increase with the number of substitutions, while the precision decreases.
