# Supplementary material for: Importance of Multiple Methylation Sites in Escherichia coli Chemotaxis
Source: PLoS One. 2015 Dec 18;10(12):e0145582. doi: 10.1371/journal.pone.0145582 (PMC4684286; doi:10.1371/journal.pone.0145582)
Supplement: S6 Fig — (PDF) [file pone.0145582.s006.pdf]

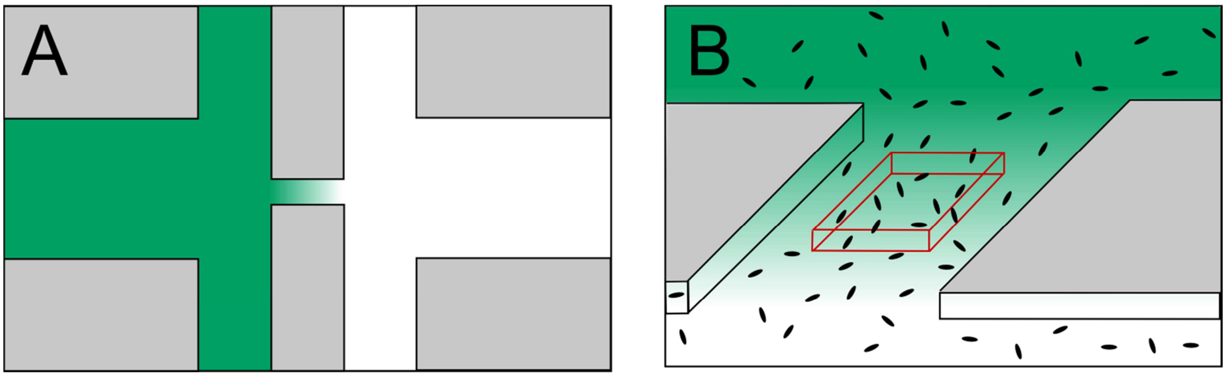

S6 Fig. Scheme of the microstructured device for liquid gradient assay. Schematic drawing of the device for measuring responses in gradients. (A) Top view of the device consisting of two reservoirs, one of which contains the attractant (MeAsp), linked by a channel in which the gradient forms. (B) 3D view of the channel in which the gradient forms. The red parallelepiped represents the observation area, in which a linear gradient of concentration forms, and for which the average concentration of attractant is half the concentration in the reservoir. Cells are brought in during the sample loading, and subsequently drift in response to the gradient.
