# Supplementary material for: Importance of Multiple Methylation Sites in Escherichia coli Chemotaxis
Source: PLoS One. 2015 Dec 18;10(12):e0145582. doi: 10.1371/journal.pone.0145582 (PMC4684286; doi:10.1371/journal.pone.0145582)
Supplement: S1 Table — (PDF) [file pone.0145582.s007.pdf]

S1 Table. EC_{50} values of different Tar mutants

Receptor	Δ*cheRcheB*		$CheR^+$ $CheB^+$	
	EC_{50}	SEM	EC_{50}	SEM
AAAA	385.5	61	303.3	74.65
EAAA	57.9	4.45	78.9	8.4
AEAA	108.8	5.43	118.5	11.5
AAEA	43.3	2.15	58.6	10.51
AAAE	68.7	9.6	117.6	9.3
AAEE	0.8	0.06	1.3	0.31
AEAE	1.3	0.21	3.5	0.46
AEEA	--	--	0.9	0.06
EAAE	--	--	4	0.96
EAEA	--	--	1.3	0.16
EEAA	52.5	4.38	53.2	3.38
AEEE	--	--	0.6	0.13
EAEE	--	--	1.3	0.4
EEAE	--	--	3	0.3
EEEA	--	--	0.6	0.12
EEEE	--	--	0.8	0.04
