# Supplementary material for: Importance of Multiple Methylation Sites in Escherichia coli Chemotaxis
Source: PLoS One. 2015 Dec 18;10(12):e0145582. doi: 10.1371/journal.pone.0145582 (PMC4684286; doi:10.1371/journal.pone.0145582)
Supplement: S2 Table — (PDF) [file pone.0145582.s008.pdf]

S2 Table. Plasmids used in this study

Plasmid	Relevant genotype	induction	reference
pVS88	*cheY-eyfp cheZ-ecfp*, pTrc99a derivative	50 μM IPTG	(47)
pVS472	tar^{AAAA}, pKG110 derivative	2 μM Sal	S. Hermann
pVS1086	tar^{EEEE}, pKG110 derivative	2 μM Sal	D. Kentner
pVS1569	tar^{AEEE}, pKG110 derivative	2 μM Sal	This work
pVS1570	tar^{EAEE}, pKG110 derivative	2 μM Sal	This work
pVS1571	tar^{EEAE}, pKG110 derivative	2 μM Sal	This work
pVS1572	tar^{EEEA}, pKG110 derivative	2 μM Sal	This work
pVS1573	tar^{AAEE}, pKG110 derivative	2 μM Sal	This work
pVS1574	tar^{AEAE}, pKG110 derivative	2 μM Sal	This work
pVS1575	tar^{AEEA}, pKG110 derivative	2 μM Sal	This work
pVS1576	tar^{EAAE}, pKG110 derivative	2 μM Sal	This work
pVS1577	tar^{EAEA}, pKG110 derivative	2 μM Sal	This work
pVS1578	tar^{EEAA}, pKG110 derivative	2 μM Sal	This work
pVS1579	tar^{AAAE}, pKG110 derivative	2 μM Sal	This work
pVS1580	tar^{AAEA}, pKG110 derivative	2 μM Sal	This work
pVS1581	tar^{AEAA}, pKG110 derivative	2 μM Sal	This work
pVS1582	tar^{EAAA}, pKG110 derivative	2 μM Sal	This work
